# Developmental Effects of the ToxCast™ Phase I and Phase II Chemicals in *Caenorhabditis elegans* and Corresponding Responses in Zebrafish, Rats, and Rabbits

**DOI:** 10.1289/ehp.1409645

**Published:** 2015-10-23

**Authors:** Windy A. Boyd, Marjolein V. Smith, Caroll A. Co, Jason R. Pirone, Julie R. Rice, Keith R. Shockley, Jonathan H. Freedman

**Affiliations:** 1 Biomolecular Screening Branch, Division of the National Toxicology Program, National Institute of Environmental Health Sciences (NIEHS), National Institutes of Health (NIH), Department of Health and Human Services (DHHS), Research Triangle Park, North Carolina, USA; 2 Social & Scientific Systems Inc., Durham, North Carolina, USA; 3 Biostatistics and Computational Biology Branch, and; 4 Laboratory of Toxicology and Pharmacology, NIEHS, NIH, DHHS, Research Triangle Park, North Carolina, USA

## Abstract

**Background:**

Modern toxicology is shifting from an observational to a mechanistic science. As part of this shift, high-throughput toxicity assays are being developed using alternative, nonmammalian species to prioritize chemicals and develop prediction models of human toxicity.

**Methods:**

The nematode Caenorhabditis elegans (C. elegans) was used to screen the U.S. Environmental Protection Agency’s (EPA’s) ToxCast™ Phase I and Phase II libraries, which contain 292 and 676 chemicals, respectively, for chemicals leading to decreased larval development and growth. Chemical toxicity was evaluated using three parameters: a biologically defined effect size threshold, half-maximal activity concentration (AC50), and lowest effective concentration (LEC).

**Results:**

Across both the Phase I and Phase II libraries, 62% of the chemicals were classified as active ≤ 200 μM in the C. elegans assay. Chemical activities and potencies in C. elegans were compared with those from two zebrafish embryonic development toxicity studies and developmental toxicity data for rats and rabbits. Concordance of chemical activity was higher between C. elegans and one zebrafish assay across Phase I chemicals (79%) than with a second zebrafish assay (59%). Using C. elegans or zebrafish to predict rat or rabbit developmental toxicity resulted in balanced accuracies (the average value of the sensitivity and specificity for an assay) ranging from 45% to 53%, slightly lower than the concordance between rat and rabbit (58%).

**Conclusions:**

Here, we present an assay that quantitatively and reliably describes the effects of chemical toxicants on C. elegans growth and development. We found significant overlap in the activity of chemicals in the ToxCast™ libraries between C. elegans and zebrafish developmental screens. Incorporating C. elegans toxicological assays as part of a battery of in vitro and in vivo assays provides additional information for the development of models to predict a chemical’s potential toxicity to humans.

**Citation:**

Boyd WA, Smith MV, Co CA, Pirone JR, Rice JR, Shockley KR, Freedman JH. 2016. Developmental effects of the ToxCast™ Phase I and II chemicals in Caenorhabditis elegans and corresponding responses in zebrafish, rats, and rabbits. Environ Health Perspect 124:586–593; http://dx.doi.org/10.1289/ehp.1409645

## Introduction

The U.S. National Toxicology Program (NTP) is charged with providing current scientific information to regulatory agencies and to the general public on the potential human health risks of environmental toxicants. Little to no toxicity information is available for thousands of chemicals that are currently in use. To address this paucity of information, the Tox21 community was established through a memorandum of understanding between the NTP, the U.S. Environmental Protection Agency (EPA), and the National Institutes of Health (NIH)Chemical Genomics Center, now the National Center for Advancing Translational Sciences (NCATS) (Collins et al. 2008). Tox21 is using high-throughput *in vitro* screening and *in vivo* alternative animal model testing to identify mechanisms of toxicity, to prioritize chemicals for additional *in vivo* toxicity testing, and to develop predictive models of human toxicological responses. As part of that effort, the U.S. EPA–National Center for Computational Toxicology (NCCT) ToxCast™ program uses batteries of *in vitro* assays in an attempt to prioritize thousands of chemicals for further toxicological testing and to develop prediction models for human toxicity (Dix et al. 2007).

The ToxCast™ Phase I library contains 292 unique chemicals, comprising mainly pesticide active ingredients (Judson et al. 2010). These chemicals are relatively well-characterized by traditional mammalian toxicity tests: data from rat and rabbit developmental toxicity tests are available for 251 and 234 of these 292 chemicals, respectively, in the U.S. EPA’s Toxicity Reference Database (ToxRefDB) (Knudsen et al. 2009). The Phase II library contains 676 unique chemicals and includes 9 chemicals from the Phase I library as well as an additional 14 replicates that function as internal tests for reproducibility. Although the chemical space is much broader for Phase II than for Phase I, given its inclusion of failed pharmaceuticals, food additives, and industrial products, many of these chemicals have not been tested in traditional mammalian assays. Human clinical data, however, are available for some of the chemical classes, such as cosmetics and failed pharmaceuticals, allowing direct linkage to human health effects (https://www.epa.gov/chemical-research/toxicity-forecaster-toxcasttm-data).

Unlike high-throughput *in vitro* assays, which can rapidly provide information on large numbers of chemicals at low cost, whole-animal models are labor intensive, time consuming, and costly, and are therefore used to test relatively small numbers of chemicals (Collins et al. 2008). Nevertheless, animal models offer certain advantages over cell-based testing models. For example, chemical effects on multiple, interacting cell types can be used to monitor a variety of phenotypic end points affected by chemical exposures (e.g., overall reproductive effects). Thus, whole-animal assays allow for the examination of complex phenotypes that often involve multiple mechanisms, and they may better represent human exposure situations.

Animal species with short developmental periods and phenotypes that can be measured using automated processes are particularly useful in rapidly estimating chemical effects on whole-organism development. The nematode *Caenorhabditis elegans* has been shown to be amenable to this process (Benson et al. 2014; Boyd et al. 2010b; Leung et al. 2011). *C. elegans* is also widely used as a model for human diseases including age-associated neurodegenerative diseases, genetic diseases, and metabolic disorders (Aitlhadj et al. 2011; Kaletta and Hengartner 2006). Previous work using *C. elegans* as a toxicological model found predictive relationships between locomotion and reproduction endpoints in *C. elegans* and lethality in rodents (Boyd et al. 2010a; Cole et al. 2004; Melstrom and Williams 2007; Williams and Dusenbery 1988).

The *C. elegans* larval growth and development assay presented in this publication provides an indication of a chemical’s effects on nematode growth and development. *C. elegans* growth, like that of many lower organisms, is not a continuous process; rather, it occurs through four distinct molts with differing sizes (Byerly et al. 1976). The assay described herein quantifies the size of individual nematodes as optical density or extinction (EXT), using a COPAS Biosort flow cytometer (Pulak 2006), after 48-hr continuous exposures to chemicals. In untreated *C. elegans*, the population at 48 hr will develop to the L4 stage such that there is a direct relationship between size and EXT. In comparison, exposed animals generally range in size and developmental stage from L1 to L4, depending on the severity of growth inhibition invoked by chemical exposures. Chemical exposures were limited to 48 hr to avoid the production of a second generation of offspring, which would complicate data analysis. Under highly toxic conditions, nematodes decrease in size or die during the 48-hr exposure (Boyd et al. 2009; Smith et al. 2009).

The goal of the present study was to determine the inhibition of *C. elegans* larval growth after exposures to the ToxCast™ Phase I and Phase II chemicals. A subset of the Phase I chemicals with known but variable growth-inhibitory potencies was first used to test the reliability and reproducibility of this assay. Optical absorption measurements were then linked with visually observed developmental stages to define a biologically relevant “effect size threshold” that was used to assess chemical activity. Because the *C. elegans* assay coincided with larval development, the *C. elegans* hazard classifications were compared with several other *in vivo* assays in which exposures occurred during the development of the animals: zebrafish embryonic development toxicity assays (Padilla et al. 2012; Truong et al. 2014) and rat and rabbit developmental toxicity assays (Sipes et al. 2011a).

## Methods

### Nematode Culture

The Bristol N2 (wild-type) strain of *C. elegans* was obtained from the *Caenorhabditis* Genetic Center and maintained at 20°C on K-agar plates (2% bacto-agar, 0.25% bacto-peptone, 51 mM sodium chloride, 32 mM potassium chloride, 13 μM cholesterol) seeded with *E. coli* OP50 as a food source (Brenner 1974; Williams and Dusenbery 1988). Age-synchronized adult nematodes were prepared using alkaline hypochlorite treatment as previously described (Khanna et al. 1997).

### Chemicals

The chemicals in the ToxCast™ Phase I and Phase II libraries (http://www.epa.gov/NCCT/toxcast/chemicals.html) were provided by the U.S. EPA in 100% dimethyl sulfoxide (DMSO), typically at concentrations of 20 mM. Because 1% DMSO did not affect *C. elegans* growth (see Figure S1), the chemicals were diluted with complete K-medium (51 mM sodium chloride, 32 mM potassium chloride, 3 mM calcium chloride, 3 mM magnesium sulfate, 13 μM cholesterol) to a maximum concentration of 200 μM. Exposures to 4% DMSO were sublethal and almost completely inhibited nematode growth (see Figure S1). Thus, 4% DMSO was used as the positive control for all experiments.

### *C. elegans* Growth Assay

Growth assays were modified from those described by Boyd et al. (2009) and employed the COPAS Biosort flow sorting system (Pulak 2006) (Union Biometrica Inc.). The Biosort was used to dispense 50 age-synchronized L1 larvae into each well of a 96-well plate containing complete K-medium, varying concentrations of the test chemical (0.5, 1, 5, 10, 50, 100, and 200 μM), 1% DMSO (final concentration), and killed OP50 *E. coli*. Nematodes were exposed to chemicals for 48 hr at 20°C, at which time untreated nematodes reached the L4 to young-adult stage (Smith et al. 2009). The Biosort was then used to measure the EXT of individual nematodes at one time immediately following 48-hr chemical exposures, and the values were converted to natural log(EXT) for analyses. Biosort measurements of extraneous material such as detritus, bacteria clumps, or precipitates were filtered from the data using a growth model, as previously described (Boyd et al. 2010b; Smith et al. 2009).

The screens of the Phase I and Phase II libraries were initiated 3 years apart (May 2008 for Phase I and May 2011 for Phase II), and the plate design was slightly altered during this time. In both screens, each 96-well plate consisted of a single concentration of eight chemicals, as well as the negative control (1% DMSO) and the positive control (4% DMSO). Additional concentrations were tested on separate 96-well plates. For Phase I, chemicals were loaded within rows with 4 wells per treatment group and rinse wells between each treatment well. For Phase II, chemicals were loaded within columns with 6 wells per treatment group followed by 2 rinse wells. Rinse wells contained 1% DMSO and were placed between treatment groups to rinse the aspiration tool and avoid carryover of animals between adjacent treatment groups. Plate adjustments were made by subtracting the mean nematode size of the plate negative controls (i.e., 1% DMSO-only–treated nematodes) for each plate, which had an average log(EXT) of 5.665, with an arbitrary value of six added for display purposes to allow a decreasing response as toxicity increased with no effect on the analysis. Subsequent analyses [lowest effective concentration (LEC) calculations, Hill function estimates, *Z*-scores, etc.] were performed using the mean size of the nematodes within an individual well after 48-hr chemical exposures.

### Classifying Chemical Activity by *C. elegans* Larval Development

**Figure 1:**
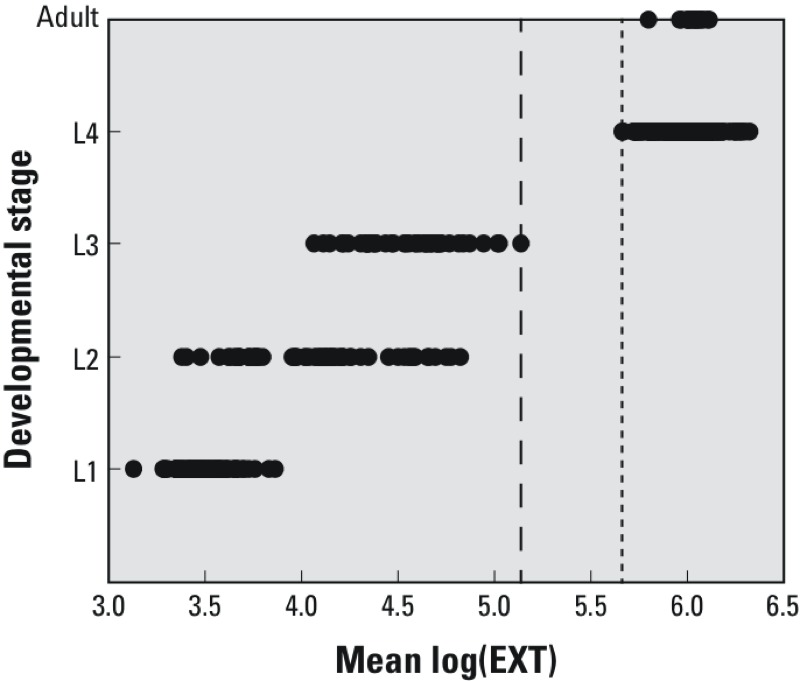
Association between *C. elegans* size and developmental stage. Nematode developmental stages (L1 larva–adult) were determined after direct observation by microscope, and then size characteristics (EXT) were measured using COPAS Biosort flow cytometry. The mean log(EXT) of the nematodes in each well for a treatment group, which contained nematodes at a single larval stage, are presented. The log(EXT) of L4s and young adults were all greater than 5.665 (dotted line); nematodes that had not developed to the L3 stage were all less than 5.138 (dashed line). Each point represents the mean size [log(EXT)] of the nematodes in an individual well.

To determine the performance characteristics of the *C. elegans* growth assay, 10 replicate plates, each of which contained eight chemicals with a wide range of growth-inhibitory effects on *C. elegans* (parathion, dichlorvos, diazinon, lindane, methyl isothiocyanate, carbaryl, isoxaben, and ethephon), were examined. Each plate contained 4 wells of each chemical at a concentration of 200 μM, in addition to negative (1% DMSO) and positive (4% DMSO) controls. The EXT values were directly linked to *C. elegans* developmental stages by performing microscopic examinations of all wells containing nematodes to determine the larval stages. The mean sizes of all nematodes [log(EXT)] within wells containing only a single larval stage were plotted against larval stage number only for Figure 1. For these analyses, 837 wells contained at least one nematode, and 432 of these wells contained larvae from only one developmental stage. Wells with mixed larvae were used in all subsequent analyses. The minimum log(EXT) value from any negative control or treatment wells containing only L4s or young adults was used as an effect size threshold. In addition, for each replicate plate, *Z*-factors were calculated as described by Zhang et al. (1999) for the 1% DMSO vehicle control samples compared with the 4% DMSO positive control samples, as well as with parathion and dichlorvos, the two most active *C. elegans* toxicants. The *Z*-factor provides a measure of assay quality by taking into account both the dynamic range and the data reliability within a single number (Zhang et al. 1999).

Active chemicals in Phase I were identified using both the effect size threshold and a weighted *t*-test, which compared log(EXT) well means from treated groups with those of the negative controls on the same plate. Both the *t*-test and the effect size threshold were used to estimate two sets of LECs for all Phase I chemicals. The [log(EXT)] values of nematodes after 48-hr exposures to each chemical were fit to a Hill function, using weighted regression with a genetic algorithm (Mullen et al. 2011) for wells having ≥ 10 nematodes. For five chemical exposures at the highest concentration (200 μM), < 10 nematodes were sampled per well. Microscopic observation revealed that all of the nematodes were dead. Because these chemicals were also active at 100 μM concentrations with ≥ 10 living nematodes per treatment well, the 200 μM data were not necessary and were excluded from toxicity estimation. The following constraints were used to prevent generation of parameter estimates outside the feasible concentration region during fitting of the Hill function: the top asymptote was constrained to be in [0, 10], the exponent in [0, 25], the AC_50_ estimate in [0, 1000], and the lower asymptote in [3.135, 10].

### Interspecies Comparisons

The *C. elegans* larval development results from the Phase I and Phase II chemical libraries were compared with those from the Zebrafish^T^ embryonic developmental assay using published LEC values (Truong et al. 2014). The results from the Phase I chemicals from the *C. elegans* larval development assay were also compared with those from the Zebrafish^P^ embryonic developmental assay using published AC_50_ estimates (Padilla et al. 2012). Two developmental summary end points for rats and rabbits from the ToxRef database (http://www.epa.gov/ncct/toxrefdb/) (Knudsen et al. 2009), “DEV_rat_Developmental” and “DEV_rabbit_Developmental,” were also compared using chemicals from the Phase I library (Sipes et al. 2011b). The outcomes given for these summary statistics are minimum LEC values over the included end points.

Outcomes among the four species were compared using performance metrics for classification of compounds as active or inactive (sensitivity, specificity, and balanced accuracy) as well as Kendall’s tau as a concordance measure. Sensitivity is the proportion of all active compounds identified as active; specificity is the proportion of all inactive compounds identified as inactive. Because a test may have either very good sensitivity or very good specificity but may not have the other, balanced accuracy (the average of sensitivity and specificity) is also calculated. Predicted active and inactive classifications were compared across the combined chemicals with results for all species, as well as within nine chemical classes identified within the Phase I library (Judson et al. 2010). Because repeated observations for the replicate chemicals in the Phase I data set were not available for mammalian or zebrafish data, comparisons between species were analyzed using averaged *C. elegans* results.

## Results

### *C. elegans* Growth Assay Performance

Eight chemicals from the Phase I library with a range of growth-inhibitory effects were selected to evaluate data quality and calibrate the range of biological effects for this assay. Mean *Z*-factors and standard deviations were calculated for these eight chemicals and for the positive control (4% DMSO). L1 and L2 stages as observed by microscopic examination were observed for the positive control, parathion, dichlorvos, and diazinon. Lindane treatments resulted in all L3 larvae for at least one replicate. Of the remaining four chemicals, treatment with methyl isothiocyanate and carbaryl resulted in mixtures of L3 and L4 larvae, and isoxaben and ethephon were similar to the negative controls: L4s and young adults only. Because *Z*-factors compare the means and standard deviations of highly toxic compounds and negative controls (Zhang et al. 1999), only the positive control and the two most toxic chemicals (parathion and dichlorvos) were used to calculate *Z*-factors. The mean *Z*-factors (± SD) relative to negative controls based on 10 replicate plates for parathion and dichlorvos were 0.779 ± 0.068 and 0.859 ± 0.034, respectively, and 0.698 ± 0.175 for the positive control (see Tables S1, S2, and S3 for the *Z*-factor data for each of the 10 replicate plates). A *Z*-factor between 0.5 and 1.0 indicates a clear separation between treated and untreated groups and is considered an “excellent assay” (Zhang et al. 1999). As indicated by the obtained mean *Z*-factors and their small standard deviations, the *C. elegans* growth assay displayed a high degree of consistency between replicate measurements with a clear separation between affected and unaffected groups.

To link measured EXT values directly to specific *C. elegans* stages of development, exposed nematodes were visually examined to determine the larval stages present. A comparison between the mean sizes [log(EXT)s] of nematodes within each well in a treatment group containing only a single larval stage and the visually observed developmental stage is presented in Figure 1. Following a 48-hr incubation, the mean log(EXT)s of L4 larvae and young adults were > 5.665, and those of L1–L3 larvae were all < 5.138. The lowest mean log(EXT), 3.135, corresponded to L1 larvae, indicating very little growth during the 48-hr exposure. Because untreated animals were L4s at the end of the exposure period, an effect size threshold was defined such that exposed nematodes with mean log(EXT) < 5.665 were considered different from controls.

### Classifying Chemical Activity on *C. elegans* Larval Development

**Figure 2:**
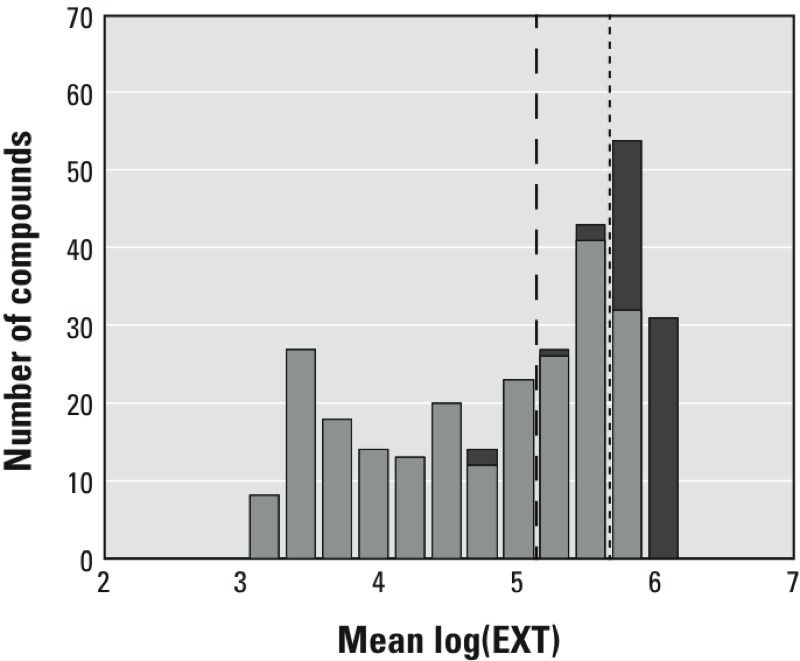
Comparison between *t*-test and effect size threshold. The histogram presents the number of chemicals in each size class [mean log(EXT)]. The dark gray bars indicate the number of inactive compounds in each size class according to the weighted *t*-test, and the light gray bars indicate the number of compounds determined to be active in each size class. The two vertical lines indicate the maximum log(EXT) for nematodes ≤ L3 (5.138) and the minimum log(EXT) (5.665) for L4 and young adult nematodes (see Figure 1). Chemicals between the vertical lines had weighted mean Log(EXT) values consistent with a mixture of L3s and L4s.

Two methods were examined for classifying the chemical activity of the compounds in the Phase I library at the highest concentration tested (200 μM): a weighted *t*-test and the effect size threshold. For the *t*-test, the mean log(EXT) values of exposed nematodes were weighted by the number of nematodes and then compared with the mean log(EXT) values of vehicle controls within the same plate. Using this method, 232 (79.5%) unique Phase I chemicals were identified as active at an overall *p* < 0.05 level (Bonferroni-corrected *p* < 0.05/292 = 0.000171232) (Figure 2; see also Excel Table S1). Using the effect size threshold of mean log(EXT) < 5.665 identified 200 chemicals as active that were also identified by the *t*-test, as well as 7 additional chemicals; 32 chemicals were identified as active only by the *t*-test. Additionally, 53 compounds were identified as inactive by both methods. Because the effect size threshold reflects the biological significance of a chemical’s growth-inhibitory effect, this method was used to classify compound activity for the remaining comparisons.

All chemicals from both the Phase I and Phase II libraries were screened at seven concentrations: 0.5, 1, 5, 10, 50, 100, and 200 μM. Two classical toxicological metrics were used to define potency: lowest effective concentrations (LECs) and half-maximal active concentrations (AC_50_s) estimated from fitting the Hill function (see Excel Table S2; Hill plots for each of the tested chemicals are available from the authors upon request). The LEC was defined as the lowest concentration at which the mean log(EXT) of the exposed nematodes was below the effect size threshold and remained below this threshold for subsequent, higher concentrations (Table 1).

**Table 1:** Phase I and Phase II chemicals with LECs at tested concentrations.

Library	Chemical concentration (μM)							
	0.5	1	5	10	50	100	200	> 200^*a*^
Phase I [*n* (percent)]	19 (6.5)	5 (1.7)	10 (3.4)	12 (4.1)	46 (15.8)	25 (8.6)	89 (30.5)	86 (29.4)
Phase II [*n* (percent)]	16 (2.4)	9 (1.3)	35 (5.2)	35 (5.2)	86 (12.7)	51 (7.5)	164 (24.3)	280 (41.4)
Total (*n*)	35	14	45	47	132	76	253	366
Cumulative total (*n*)	35	49	94	141	273	349	602	968^*b*^
^***a***^LEC > 200 indicates a compound that may affect nematode growth above the tested concentrations 0.5–200 μM. These compounds may also be inactive at any concentration. ^***b***^Nine chemicals are replicated in the Phase I and II libraries, so 959 unique chemicals across both libraries.								

### Interspecies Comparisons of Toxicity: ToxCast™ Phase I

**Figure 3:**
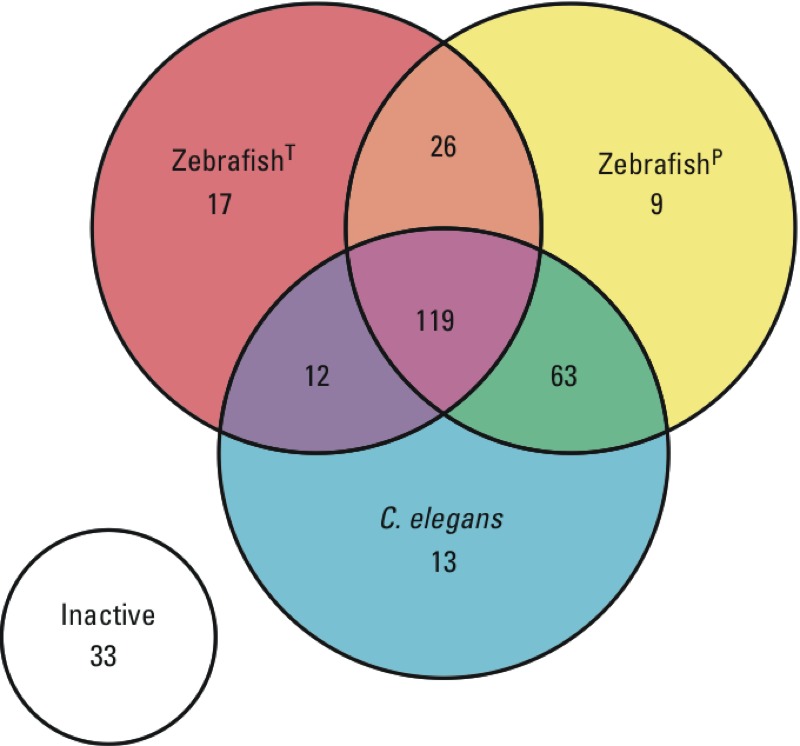
Concordance between *C. elegans* larval development and zebrafish embryonic development assays for ToxCast™ Phase I chemical activity. Venn diagram illustrating the concordance between the effects of chemicals on *C. elegans* development and two zebrafish development assays, Zebrafish^P^ (Padilla et al. 2012) and Zebrafish^T^ (Truong et al. 2014).

*Comparison with zebrafish development.* The *C. elegans* results for the Phase I chemical library were compared with those from two zebrafish embryo developmental assays referred to as Zebrafish^P^ (Padilla et al. 2012) and Zebrafish^T^ (Truong et al. 2014). Zebrafish^P^ estimated AC_50_s and AC_10_s using a composite deformity score after chemical exposures at concentrations ranging from 1 nM to 80 μM, whereas Zebrafish^T^ estimated LECs across 18 end points, including mortality, after exposure to chemicals at concentrations ranging from 6.4 nM to 64 μM. The minimum LEC calculated from all 18 zebrafish embryonic development end points was used for comparison with *C. elegans* data. Of 292 unique chemicals, there was agreement among all three assays for 152 compounds: 119 active and 33 inactive (Figure 3), for a concordance of 0.52. The two zebrafish assays were in accord for 191 chemicals (145 active, 46 inactive), with a concordance of 0.65; Zebrafish^P^ results were in accord with the *C. elegans* results for 232 chemicals (182 active, 50 inactive), with a concordance of 0.79; and Zebrafish^T^ results were in accord with the *C. elegans* results for 173 chemicals (131 active, 42 inactive), with a concordance of 0.59. The potency ranks of the Phase I chemicals were also compared between *C. elegans* and the two zebrafish assays. When 122 AC_50_s with estimates less than the maximum tested concentration were compared between *C. elegans* and Zebrafish^P^ (see Excel Table S2), a nonsignificant correlation of 0.078 was estimated by Kendall’s tau (*p* = 0.40). When LEC values were compared between Zebrafish^T^ and *C. elegans*, a slight, but significant, correlation was estimated (Kendall’s tau = 0.108; *p* = 0.021).

*Comparison with mammalian development.* The U.S. EPA’s Toxicity Reference Database (ToxRefDB) (Martin et al. 2009) contains summary statistics consisting of minimum LECs for 27 developmental outcomes for rats and 26 developmental outcomes for rabbits exposed to most of the Phase I chemicals (DEV_rat_Developmental and DEV_rabbit_Developmental, respectively) (Sipes et al. 2011a). Composite LECs for the rabbit and rat developmental end points were available for 234 and 251 chemicals, respectively. A chemical was classified as inactive for these outcomes if it was tested, but no LEC was reported. The rat and rabbit composite LECs were compared with LECs and AC_50_s from *C. elegans* and with the two zebrafish embryonic development assays. For the 200 chemicals tested in all species, the percent active chemicals in the Phase I library were 71% for C. *elegans*, 75% for Zebrafish^P^, 61% for Zebrafish^T^, 43% for rabbits, and 59% for rats. Balanced accuracy estimates (the average of sensitivity and specificity) for predicting rat and rabbit developmental toxicity based on *C. elegans* assays were 53% and 52%, respectively, compared with corresponding estimates of 52% (Zebrafish^P^) and 51% (Zebrafish^T^) for rat and 45% (Zebrafish^P^) and 50% (Zebrafish^T^) for rabbit by the two zebrafish assays (Table 2). *C. elegans* assays were the most sensitive for rabbit toxicity (74% compared with 60–68%), and Zebrafish^P^ assays were the most sensitive for rat toxicity (76% compared with 61–74%). *C. elegans* assays were the most sensitive for predicting rabbit toxicity [74% compared with 60% (Zebrafish^T^) and 68% (Zebrafish^P^)], and Zebrafish^P^ assays were the most sensitive for rat toxicity [76% compared with 61% (Zebrafish^T^) and 74% (*C. elegans*)]. The specificities of *C. elegans* assays for predicting rabbit and rat toxicity were 30% and 32% respectively, and corresponding values for the zebrafish assays were 21% (Zebrafish^P^) and 39% (Zebrafish^T^) for rabbit, and 28% (Zebrafish^P^) and 40% (Zebrafish^T^) for rat. The concordance between rat and rabbit development was 58%, with 59/200 active and 56/200 inactive for both.

**Table 2:** Accuracy of *C. elegans* or zebrafish embryogenesis toxicity data for predicting developmental outcomes in rabbits and rats.

Predicting species	Predicted species^*a*^	
	Rabbit	Rat
*C. elegans*
BA (percent)	52.3	52.7
Sensitivity (percent)	74.1	73.7
Specificity (percent)	30.4	31.7
Zebrafish^P^
BA (percent)	44.6	52.2
Sensitivity (percent)	68.2	76.3
Specificity (percent)	20.9	28.0
Zebrafish^T^
BA (percent)	49.6	50.6
Sensitivity (percent)	60.0	61.0
Specificity (percent)	39.1	40.2
BA, balanced accuracy = average of sensitivity and specificity. Data were available across all species for 200 unique chemicals. Zebrafish^P^ is from Padilla et al. (2012), and Zebrafish^T^ is from Truong et al. (2014). ^***a***^The species listed in each row was used to predict the outcome of the species across columns.		

*Comparison by chemical class.* The activities of the Phase I chemicals within previously described chemical classes (Judson et al. 2010) were assessed in *C. elegans*, zebrafish, rat, and rabbit development (Table 3 and Table 4). The most active chemical class across species was conazoles, with the lowest number of active chemicals observed in rabbit. Amides, anilides, and organophosphates had a higher percentage of active chemicals in nematodes and zebrafish than in rats and rabbits. Overall, Zebrafish^P^ had the highest proportion of active chemicals, followed by *C. elegans* and then Zebrafish^T^, whereas rabbit had the lowest proportion of actives.

**Table 3:** Proportion of chemicals classified as active and concordance between assays among groups of Phase I chemicals.

Chemical class^*a*^ (number of chemicals)	Proportion active^*b*^			Concordance^*c*^		
	*C. elegans*	Zebrafish^P^	Zebrafish^T^	*C. elegans* and Zebrafish^P^	*C. elegans* and Zebrafish^T^	Zebrafish^P^ and Zebrafish^T^
Amide (24)	0.58	0.75	0.75	0.75	0.50	0.75
Anilide (14)	0.64	0.79	0.86	0.86	0.50	0.64
Carbamate (15)	0.53	0.80	0.67	0.60	0.73	0.73
Conazole (18)	1.00	1.00	0.89	1.00	0.89	0.89
Organophosphate (35)	0.80	0.86	0.57	0.83	0.49	0.54
Phenoxy (12)	0.67	0.92	0.33	0.75	0.33	0.42
Pyrethroid (12)	0.92	1.00	0.67	0.92	0.58	0.67
Pyridine (10)	0.70	0.60	0.40	0.90	0.50	0.60
Urea (8)	0.63	0.75	0.63	0.38	0.50	0.38
^***a***^Chemical classes were derived from Judson et al. (2010). ^***b***^Chemical activity is based on the specific assays for Zebrafish^P^ (Padilla et al. 2012), Zebrafish^T^ (Truong et al. 2014), and *C. elegans* (this publication). ^***c***^Concordance is defined as the proportion of chemicals with the same classification.						

**Table 4:** Balanced accuracy*^a^* of *C. elegans*, Zebrafish^P^, and Zebrafish^T^ assays for predicting developmental outcomes in rabbits and rats according to chemical class.

Chemical class	Rats^*b*^					Rabbits^*b*^				
	*n*	Percent active	*C. elegans*	Zebrafish^P^	Zebrafish^T^	*n*	Percent active	*C. elegans*	Zebrafish^P^	Zebrafish^T^
Amide	21	0.48	0.62	0.63	0.54	22	0.36	0.76	0.72	0.42
Anilide	14	0.50	0.57	0.57	0.50	14	0.43	0.81	0.69	0.33
Carbamate	14	0.71	0.50	0.70	0.43	14	0.50	0.64	0.71	0.71
Conazole	16	1.00	All active^*c*^	All active^*c*^	2 inactive^*c*^	16	0.69	0.50	0.50	0.41
Organophosphate	25	0.32	0.43	0.53	0.36	25	0.24	0.50	0.58	0.60
Phenoxy	8	0.75	0.50	0.75	0.75	11	0.27	0.52	0.33	0.31
Pyrethroid	12	0.50	0.42	0.50	0.33	10	0.40	0.38	0.50	0.33
Pyridine	7	0.43	0.63	0.75	0.42	6	0.50	0.33	0.50	1.00
Urea	6	0.83	0.30	0.30	0.90	5	0.60	0.75	0.17	0.75
^***a***^Average sensitivity and specificity. ^***b***^Data for rats and rabbits were obtained from the ToxRef database (http://www.epa.gov/ncct/toxrefdb/) (Knudsen et al. 2009). ^***c***^Unable to calculate balanced accuracy because of the absence of sufficient negative results.										

The concordance between *C. elegans* growth and the two zebrafish embryonic development assays within Phase I chemical classes is presented in Table 3. As observed for all of the Phase I chemicals, the *C. elegans* growth results agree well with those of Zebrafish^P^ across most of the chemical categories. However, although similar numbers of urea chemicals were active in both assays, the concordance was only 38%: *C. elegans* indicated five active and three inactive, Zebrafish^P^ identified six active and two inactive, but only three of eight chemicals received the same classification from both assays (Table 3). The concordance between *C. elegans* and Zebrafish^T^ was highest for conazoles, carbamates, and pyrethroids, and was otherwise ≤ 50%. The concordance between the two zebrafish assays was < 50% for the phenoxy and urea chemical classes.

Finally, the *C. elegans* and two zebrafish assay results were used to predict activity in rat and rabbit development within chemical classes using balanced accuracy estimates (Table 4). Overall, zebrafish and *C. elegans* predictions of mammalian outcomes were similar within most chemical classes. The balanced accuracies for prediction of rabbit development using *C. elegans* growth were highest for anilide (0.81), amide (0.76), and urea (0.75), whereas all of the balanced accuracies for prediction of rat from *C. elegans* were ≤ 0.70. For Zebrafish^P^, balanced accuracies for rat were highest for phenoxy (0.75), pyridine (0.75), and carbamate (0.70) classes; and these values were highest for rabbit: amide (0.72) and carbamate (0.71). Balanced accuracies for Zebrafish^P^ were lowest for urea compounds (0.30 in rats and 0.17 in rabbits), but highest for Zebrafish^T^ (0.90 in rats and 0.75 in rabbits). The combined sensitivity and specificity of *C. elegans* assays for urea compounds was low for rats (0.30) and comparable to that of Zebrafish^T^ for rabbits (0.75).

### Combined ToxCast™ Phase I and Phase II

**Figure 4:**
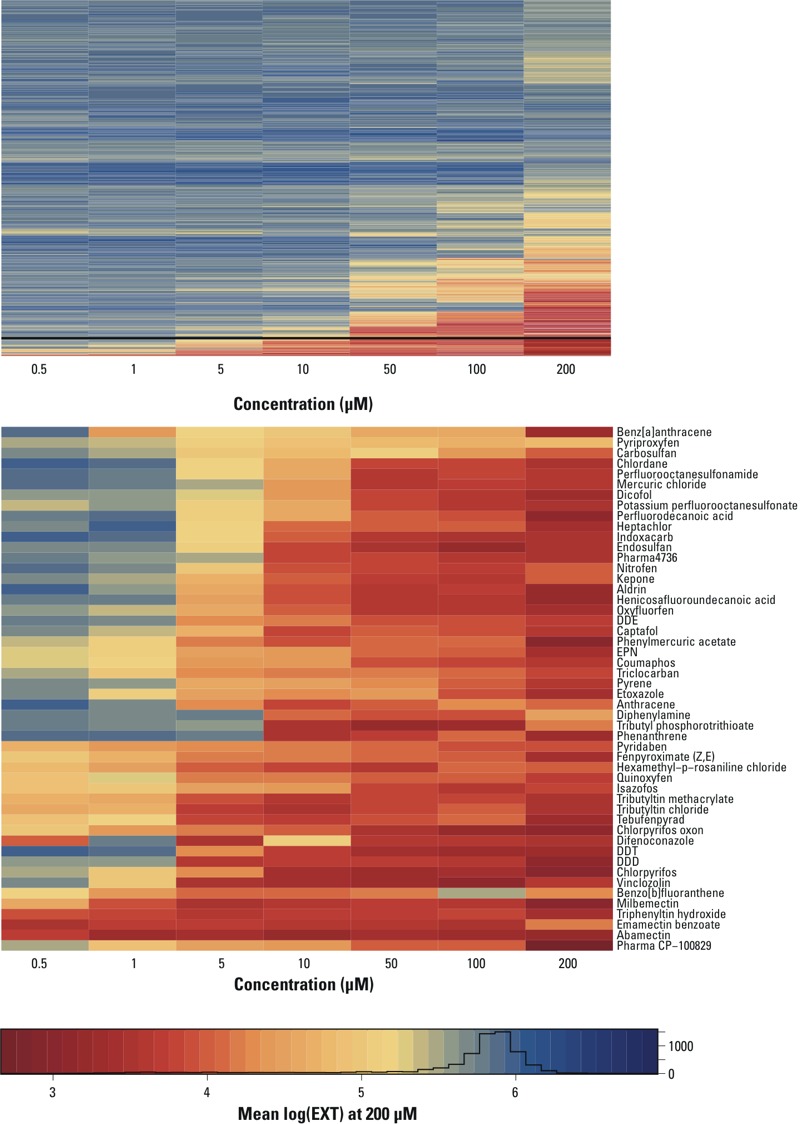
Hierarchical clustering of chemical activity on *C. elegans* development. Blue corresponds to inactive chemicals with responses similar to controls, and yellow to red indicates decreasing nematode size with increasing toxicity. The histogram illustrates the size distribution of matched negative controls. Upper panel: activity of 959 unique chemicals from ToxCast™ Phase I and Phase II libraries clustered according to mean log(EXT). Lower**panel: activity and chemical names of the 50 chemicals with the greatest effects on *C. elegans* growth. Lists and descriptions of chemicals in the lower panel are presented in Table S4.

*Activity in* C. elegans *larval growth and development assay.* In Figure 4, the 959 unique chemicals from the combined Phase I and Phase II libraries are clustered using the mean log(EXT) for the *C. elegans* assay at all concentrations tested. Overall, the number of active chemicals and the intensity of effects monotonically increased with concentration. The 50 chemicals with the greatest effect on growth at the highest concentration tested (200 μM) comprised mainly pesticides and included several organophosphates [chlorpyrifos, chlorpyrifos oxon, isazofos, coumaphos, O-ethyl O-(4-nitrophenyl) phenylphosphonothioate (EPN)], organotins (triphenyltin hydroxide, tributyltin chloride, and tributyltin methacrylate), avermectins (abamectin, emamectin benzoate, and milbemectin), and organochlorines {1-chloro-4-[2,2-dichloro-1-(4-chlorophenyl)ethyl]benzene (DDD), 1-chloro-4-[2,2,2-trichloro-1-(4-chlorophenyl)ethyl]benzene (DDT), 1-chloro-4-[2,2-dichloro-1-(4-chlorophenyl)ethenyl]benzene (DDE), and dicofol}. Nineteen of the 50 chemicals were also active at the lowest concentration tested (0.5 μM) (see Excel Table S2 and Table S4); these chemicals, listed by increasing mean log(EXT) at 0.5 μM, are emamectin benzoate, abamectin, fentin, milbemectin, pyridaben, isazofos, quinoxyfen, tebufenpyrad, chlorpyrifos oxon, fenpyroximate, coumaphos, methylene bis(thiocyanate), molinate, fenamiphos, pyriproxyfen, oxyfluorfen, parathion, methoxychlor, and dicofol.

*Replicate analysis.* Replicate chemicals were included by the ToxCast™ program in each library to monitor assay performance (Table 5). The Phase I library included four chemicals replicated twice [3-iodo-2-propynylbutylcarbamate (IPBC), dibutyl phthalate (DBP), *S*-ethyl dipropylthiocarbamate (EPTC), and fenoxaprop-ethyl] and two that were replicated three times (bensulide and diclofop-methyl), and the Phase II library contained seven chemicals from the Phase I library replicated three times [allethrin, azoxystrobin, bisphenol A, oryzalin, perfluorooctane sulfonic acid (PFOS), triadimenol, and triclosan] and two additional chemicals from Phase I that were replicated six times (clorophene and mancozeb). Chemicals with LECs or AC_50_s ≤ 200 μM were classified as active, and those with neither LEC nor AC_50_ were classified as inactive. Most of the chemicals were classified as active in all replicate samples except for EPTC, which was inactive in both replicates. In two cases, the results were not in accord across all replicates: mancozeb was inactive when tested with the Phase I library but was active in all six replicates within the Phase II library; and triadimenol was active in two replicates and inactive in the other two. In both cases, the chemicals were weakly active even at 200 μM, as indicated by mean sizes [represented by log(EXT) at 200 μM] near the size effect threshold of 5.665. In contrast with classification as active or inactive, LEC and AC_50_ values varied among the replicate samples.

**Table 5:** Replicate concordance among chemicals in the Phase I and Phase II libraries.

Chemical	Phase	Log(EXT) at 200 μM	LEC	AC_50_	Hazard^*a*^
Allethrin	I	5.37	50	NC	Active
	II	5.39	100	NC	Active
	II	4.93	50	NC	Active
	II	5.22	200	NC	Active
Azoxystrobin	I	5.51	200	195.8	Active
	II	5.60	200	NC	Active
	II	5.43	200	196.5	Active
	II	5.44	50	NC	Active
Bensulide	I	3.71	50	16.3	Active
	I	3.83	50	13.7	Active
	I	3.49	100	79.8	Active
Bisphenol A	I	5.37	200	NC	Active
	II	5.57	200	NC	Active
	II	5.38	200	NC	Active
	II	5.52	200	NC	Active
Clorophene	I	3.61	200	68.8	Active
	II	3.87	10	160.6	Active
	II	3.83	50	57.6	Active
	II	3.65	50	84.9	Active
	II	3.92	0.5	80.9	Active
	II	3.79	50	113.6	Active
	II	3.91	50	39.4	Active
Dibutyl phthalate	I	5.58	200	NC	Active
	I	5.26	50	21.3	Active
Diclofop-methyl	I	4.92	200	179.0	Active
	I	4.46	50	179.3	Active
	I	4.47	50	56.2	Active
EPTC	I	6.02		NC	Inactive
	I	5.70		NC	Inactive
Fenoxaprop-ethyl	I	5.01	100	76.7	Active
	I	5.36	50	46.0	Active
IPBC	I	3.00	200	138.7	Active
	I	3.34	100	74.3	Active
Mancozeb	I	5.75		NC	Inactive
	II	5.35	200	NC	Active
	II	5.37	200	NC	Active
	II	5.24	100	NC	Active
	II	5.47	0.5	124.1	Active
	II	5.24	100	NC	Active
	II	5.29	200	NC	Active
Oryzalin	I	3.97	50	136.3	Active
	II	4.95	50	19.6	Active
	II	4.72	10	49.9	Active
	II	4.57	10	NC	Active
PFOS	I	3.66	200	177.3	Active
	II	3.06	5	18.5	Active
	II	3.22	0.5	13.5	Active
	II	3.39	5	6.1	Active
Triadimenol	I	4.99	200	189.4	Active
	II	5.63	200	NC	Active
	II	5.94		NC	Inactive
	II	5.79		NC	Inactive
Triclosan	I	3.98	50	109.6	Active
	II	3.83	10	69.1	Active
	II	4.06	50	43.2	Active
	II	4.15	10	26.3	Active
Abbreviations: AC_50_, half-maximal activity concentration; EPTC, *S*-ethyl dipropylthiocarbamate; EXT, extinction; IPBC, 3-iodo-2-propynyl *N*-butylcarbamate; LEC, lowest effective concentration; NC, could not be calculated; PFOS, 1,1,2,2,3,3,4,4,5,5,6,6,7,7,8,8,8-heptadecafluorooctane-1-sulfonic acid. ^***a***^Chemicals were classified as active if they had an LEC or AC_50_ ≤ 200 μM, otherwise they were classified as inactive.					

*Comparison with zebrafish development.* Combined results for Phase I and Phase II chemicals were available for *C. elegans* and Zebrafish^T^. Of the 959 unique chemicals, the two assays were in accord for 560 chemicals (363 active and 197 inactive) for a concordance of 0.58. Zebrafish^T^ classified 167 chemicals as active that were inactive in the *C. elegans* assay, and 232 chemicals were active based on the *C. elegans* assay but were inactive based on Zebrafish^T^. Kendall’s tau was used to compare LECs by rank and was estimated to be 0.102 (*p* = 0.000097). Considering only the 603 compounds for which Zebrafish^T^ mortality occurred at a higher concentration than the first teratogenic effect or did not occur at all (Truong et al. 2014), the nematode and zebrafish assay results were in accord for 314 compounds (117 active and 197 inactive) for a concordance of 0.52.

## Discussion

The present study presents a high-throughput whole-animal screen using the nematode *C. elegans*. *C. elegans* and other *in vivo* animal models offer many benefits over cell-based models for the prediction of human toxicological responses. However, the ability of any animal model, from nematodes to mammals, to respond in a manner similar to humans is limited by how well the organism and the toxicological assays replicate human exposure conditions (stage of development, route of exposure, etc.) and cellular, biochemical, and molecular responses. Like all *in vivo* models, *C. elegans* contains many processes similar to those of higher organisms (Shaye and Greenwald 2011), but it is deficient in others. Although *C. elegans* cannot replicate all of the processes necessary to predict the effects of all compounds in humans, its level of homology with humans is sufficient to include it with other *in vivo* models in predictive toxicology and in the development of adverse outcome pathways. A thorough review of conserved toxicity pathways is available in the 2000 National Research Council Committee on Developmental Toxicology report [National Research Council (NRC) 2000].

The *C. elegans* automated assay uses COPAS Biosort flow cytometry to screen for the effects of chemicals on C. *elegans* larval growth and development. The results presented in this paper show that the C. *elegans* growth assay produced excellent *Z*-scores with values for the positive control and for two active chemicals that ranged between 0.5 and 1 (Zhang et al. 1999), and the consistency of the responses across 10 replicates indicated that the assay produced responses to chemicals that were highly reproducible and distinguishable from untreated controls. The assay also produced reliable hazard identification at the highest concentration tested across replicate chemicals within the ToxCast™ Phase I and Phase II libraries (Table 5).

Two methods were applied to classify chemical activity: a statistical *t*-test and a newly defined effect size threshold (Figure 1). The statistical *t*-test determined the difference between exposed and control groups, incorporating variability of the samples and providing *p*-values. The low variability within the samples, however, led to a number of compounds being classified as having statistically significant effects on growth, even though little difference in size was measured. Because relatively few compounds that induced growth inhibition were classified as inactive by the *t*-test, the effect size threshold was used for the remainder of the analysis (Figure 2). Thus, if the mean log(EXT) of exposed nematodes was below the effect size threshold, the chemical was classified as active.

Nineteen chemicals were classified as most active by hierarchical clustering of the effect size (Figure 4) and were active at the lowest concentration tested (0.5 μM) (see Table S4 and Excel Table S2). Unsurprisingly, several avermectins, which are pesticides that are used primarily to control parasitic nematodes, mites, fleas, and other insects, were classified as active. Two of the avermectins most toxic to *C. elegans*, emamectin benzoate and abamectin, were potent inhibitors of development in both Zebrafish^P^ and Zebrafish^T^ and have also been shown to be potent inhibitors of spontaneous movement in zebrafish embryos, indicating potential developmental neurotoxic effects (Raftery et al. 2014). A number of other compounds that are known or suspected developmental neurotoxicants in a variety of *in vitro* and *in vivo* models (Crofton et al. 2011; Grandjean and Landrigan 2014) were also among the most toxic chemicals to *C. elegans* in this study, including the organophosphate chlorpyrifos and its metabolite chlorpyrifos oxon, the organochlorine DDT and its metabolites, two tributyltin compounds and triphenlytin, and several polyaromatic hydrocarbons (PAHs) (see Table S4).

The results of two different zebrafish embryonic development assays were compared with the *C. elegans* results: the Zebrafish^P^ assay (Padilla et al. 2012), with results for only the Phase I chemicals, and the Zebrafish^T^ assay (Truong et al. 2014), with results for Phase I and Phase II chemicals. We note that although both the *C. elegans* and Zebrafish^P^ assays determined activity on the basis of severity of treatment effects, the Zebrafish^T^ assay determined activity on the basis of incidence of treatment effects. Other major differences in experimental design between the two studies included the presence or absence of the acellular chorion, repeated versus static exposures, and manual versus automated morphometric analyses. Overall, the *C. elegans* larval development assay was found to be in excellent agreement with the Zebrafish^P^ embryo development assay, with a concordance of nearly 80% for the Phase I chemicals, whereas concordance of the *C. elegans* larval development assay with the Zebrafish^T^ assay was lower (59% for Phase I and 58% for Phase I and Phase II).

Both the C. *elegans* and zebrafish assays describe developmental effects of chemical exposures; therefore, the responses in these species were compared with developmental effects indices for rats and rabbits in ToxRefDB for the 200 Phase I chemicals tested in all four species. By combining a suite of developmental outcomes into a single value within each species (i.e., rat and rabbit)(Sipes et al. 2011a), the numbers of active and inactive chemicals, as identified by these two indices, were reasonably well balanced. However, a clear pattern of chemical activity prediction did not emerge. Although the Zebrafish^P^ and *C. elegans* assays did have high concordance, neither predicted classification of activity in either rabbits or rats (combined average sensitivity and specificity, ~50%, Table 2). Although the balanced accuracies for these assays were similar to those for Zebrafish^T^, the concordance was much lower. Again, this discrepancy was likely due to the measurement of incidence in the Zebrafish^T^ studies versus the measurement of severity of response in the rat and rabbit studies. The rat and rabbit studies did provide some information for each other, but with lower concordance than might have been expected (~58%).

Interestingly, the poor performance of the two predictor species (*C. elegans* and zebrafish) was not uniform across chemical classes within the Phase I library (Table 3 and Table 4). When predictions were evaluated within chemical classes (Table 4), the balanced accuracy ranged from a high of 81% (*C. elegans* predicting rabbit end points for anilide compounds) to a low of 17% (Zebrafish^P^ predicting rabbit toxicity for urea compounds). When Phase I and Phase II chemical activity at each concentration were grouped using hierarchical clustering, chemicals within chemical classes appeared to cluster together (Figure 4; see also Table S4). Taken together, the large disparity in predictive powers between chemical classes and the clustering of activity suggest that quantitative structure–activity relationship (QSAR) methods could play a large role in the eventual predictive battery of assays.

Throughout the results presented in this paper, the estimation or prediction of potency was found to be less reliable than identification or concordance of chemical activity. Table 5 shows response estimates [i.e., mean size or log(EXT)] at the high concentration to be very consistent across replicates, whereas the AC_50_ estimates vary to a much greater extent. In cross-species comparisons, although the concordance of *C. elegans* active predictions with those of Zebrafish^P^ was quite good at 0.79, no significant correlation was found between chemical potencies (Kendall’s tau coefficient 0.078; *p* = 0.40).

## Conclusions

Here, we present an assay that quantitatively and reliably describes the effects of chemical toxicants on *C. elegans* growth and development. We found substantial overlap in the activity of chemicals in the ToxCast™ Phase I library in the Zebrafish^P^ and *C. elegans* developmental screens, but lower concordance was found between the *C. elegans* and Zebrafish^T^ developmental screens for the combined Phase I and Phase II libraries. Prediction of mammalian effects from *C. elegans* or zebrafish responses was poor across the Phase I library but was higher within certain chemical class–assay combinations. Incorporating other *C. elegans* toxicological assays, such as feeding (Boyd et al. 2007) and reproduction (Boyd et al. 2010a), could provide additional insights into the specificity of end points and yield further information that would add to the overall utility of *C. elegans* as an alternative toxicological model. We propose using *C. elegans* assays as part of a battery of toxicity tests and analytical methods including *in silico* modeling and prediction, cell-free and cell-based *in vitro* assays, alternative toxicological model organisms such as zebrafish and *Daphnia*, traditional toxicological model organisms such as rodents and rabbits, and relevant human data, including clinical and epidemiological observations.

## Supplemental Material

(354 KB) PDF

(259 KB) ZIP
