# Supplementary material for: Developmental Effects of the ToxCast™ Phase I and Phase II Chemicals in Caenorhabditis elegans and Corresponding Responses in Zebrafish, Rats, and Rabbits
Source: Environ Health Perspect. 2015 Oct 23;124(5):586–93. doi: 10.1289/ehp.1409645 (PMC4858399; doi:10.1289/ehp.1409645)
Supplement: (354 KB) PDF [file ehp.1409645.s001.acco.pdf]

Environ Health Perspect
DOI: 10.1289/ehp.1409645

Note to readers with disabilities: *EHP* strives to ensure that all journal content is accessible to all readers. However, some figures and Supplemental Material published in *EHP* articles may not conform to 508 standards due to the complexity of the information being presented. If you need assistance accessing journal content, please contact ehp508@niehs.nih.gov. Our staff will work with you to assess and meet your accessibility needs within 3 working days.

Supplemental Material

Developmental Effects of the ToxCast™ Phase I and II Chemicals in *Caenorhabditis elegans* and Corresponding Responses in Zebrafish, Rats, and Rabbits

Windy A. Boyd, Marjolein V. Smith, Caroll A. Co, Jason R. Pirone, Julie R. Rice, Keith R. Shockley, and Jonathan H. Freedman

Table of Contents

Table S1. Estimated Z-factors and summary statistics using 4% DMSO with vehicle control

Table S2. Estimated Z-factors and summary statistics using parathion

Table S3. Estimated Z-factors and summary statistics using dichlorvos

Table S4. Description of chemicals in the lower panel of Figure 4

Figure S1. Effect of DMSO (vehicle control) on C. elegans growth. The red dashed line indicates the AC_{50} (half-maximal concentration; 3.1 % DMSO); the blue dashed line indicates the benchmark concentration lower limit (1.6 % DMSO), below which no effect was observed compared to the untreated control. The benchmark concentration was calculated using mean nematode sizes for exposure wells, with size at time = 0 subtracted from the size at t=48 h. The definition of Crump (2002) was used to calculate the BMC with bootstrapping on residuals for the 90% one-sided confidence interval as previously described (Boyd et al. 2010a; Crump 2002).

Supplemental Code and Data Zip File

Excel Table S1. ToxCast Phase I mean log(EXT) values and weighted t-test Bonferoni-corrected p-values

Excel Table S2. Phase I and II *C. elegans* and zebrafish toxicity estimates

Table S1. Estimated Z-factors and summary statistics using 4% DMSO with vehicle control.

Plate Number	Z-factor	Standard Deviation (negative control)	Standard Deviation (positive control)	Mean (negative control)	Mean (positive control)
**1**	0.8113	0.0628	0.0640	5.8411	3.8252
**2**	0.3960	0.0572	0.3016	5.8322	4.0496
**3**	0.8762	0.0551	0.0416	5.9068	3.5642
**4**	0.8264	0.0618	0.0655	5.7675	3.5671
**5**	0.4678	0.0446	0.2511	5.7687	4.1021
**6**	0.8683	0.0295	0.0780	5.7737	3.3251
**7**	0.7080	0.0606	0.0669	5.8553	4.5454
**8**	0.6139	0.0771	0.0961	5.7169	4.3707
**9**	0.8395	0.0330	0.0775	5.8052	3.7388
**10**	0.5768	0.1079	0.2145	5.9039	3.6183

Using the 10 replicate plates in the preliminary experiment, the Z-factor and summary statistics were calculated using 4% DMSO as positive control. The summary statistics include standard deviations and means for the negative control (1% DMSO) as well as the positive control (4% DMSO).

Table S2. Estimated Z-factors and summary statistics using parathion.

Plate Number	Z-factor	Standard Deviation (negative control)	Standard Deviation (positive control)	Mean (negative control)	Mean (positive control)
**1**	0.7620	0.0628	0.1227	5.8411	3.5036
**2**	0.7310	0.0572	0.1557	5.8322	3.4572
**3**	0.8292	0.0551	0.0827	5.9068	3.4870
**4**	0.8065	0.0618	0.0895	5.7675	3.4228
**5**	0.7546	0.0446	0.1530	5.7687	3.3531
**6**	0.8618	0.0295	0.0801	5.7737	3.3938
**7**	0.7740	0.0606	0.1140	5.8553	3.5372
**8**	0.6805	0.0771	0.1814	5.7169	3.2893
**9**	0.8894	0.0330	0.0496	5.8052	3.5646
**10**	0.7020	0.1079	0.1360	5.9039	3.4490

Using the 10 replicate plates in the preliminary experiment, the Z-factor and summary statistics were calculated using 200 µM parathion as positive control. The summary statistics include standard deviations and means for the negative control (1% DMSO) as well as the positive control (Parathion).

Table S3. Estimated Z-factors and summary statistics using dichlorvos.

Plate Number	Z-factor	Standard Deviation (negative control)	Standard Deviation (positive control)	Mean (negative control)	Mean (positive control)
**1**	0.8676	0.0628	0.0513	5.8411	3.2570
**2**	0.8565	0.0572	0.0657	5.8322	3.2618
**3**	0.8720	0.0551	0.0576	5.9068	3.2649
**4**	0.8309	0.0618	0.0786	5.7675	3.2781
**5**	0.8874	0.0446	0.0521	5.7687	3.1920
**6**	0.8713	0.0295	0.0772	5.7737	3.2864
**7**	0.8477	0.0606	0.0617	5.8553	3.4451
**8**	0.8431	0.0771	0.0551	5.7169	3.1873
**9**	0.9167	0.0330	0.0359	5.8052	3.3231
**10**	0.7922	0.1079	0.0746	5.9039	3.2689

Using the 10 replicate plates in the preliminary experiment, the Z-factor and summary statistics were calculated using 200 µM dichlorvos, as positive control. The summary statistics include standard deviations and means for the negative control (1% DMSO) as well as the positive control (Dichlorvos).

Table S4. Description of chemicals in the lower panel of Figure 4.

Order (1-most toxic)	CASRN	Name in Figure 4	Chemical name	Classification
1	135080-03-4	Pharma CP-100829	6-chloro-3-[(4-chlorothiophen-2-yl)carbonyl]-5-fluoro-2-oxo-2,3-dihydro-1H-indole-1-carboxamide	failed pharmaceutical
2	71751-41-2	Abamectin	Abamectin	avermectin
3	155569-91-8	Emamectin benzoate	Emamectin benzoate	avermectin
4	76-87-9	Triphenyltin hydroxide	Fentin	organotin
5	NOCAS	Milbemectin	Milbemectin (mix of >70% Milbemycin A4 CAS 51596-11-3; <30% Milbemycin A3 CAS 51596-10-2)	avermectin
6	205-99-2	Benzo[b]fluoranthene	Benzo[b]fluoranthene	PAH
7	50471-44-8	Vinclozolin	Vinclozolin	fungicide/antiandrogenic
8	2921-88-2	Chlorpyrifos	Chlorpyrifos	OP pesticide
9	72-54-8	DDD	p,p'-DDD	OC metabolite
10	50-29-3	DDT	p,p'-DDT	OC pesticide
11	119446-68-3	Difenoconazole	Difenoconazole	fungicide
12	5598-15-2	Chlorpyrifos oxon	Chlorpyrifos oxon	OP metabolite
13	119168-77-3	Tebufenpyrad	Tebufenpyrad	pyrazole insecticide
14	1461-22-9	Tributyltin chloride	Tributyltin chloride	organotin
15	2155-70-6	Tributyltin methacrylate	Stannane, tributyl(2-methyl-1-oxo-2-propenyl)oxy-	organotin
16	42509-80-8	Isazofos	Isazofos	OP pesticide
17	124495-18-7	Quinoxyfen	Quinoxyfen	fungicide
18	548-62-9	Hexamethyl-p-rosaniline chloride	Hexamethyl-p-rosaniline chloride	crystal violet
19	111812-58-9	Fenpyroximate (Z,E)	(Z,E)-Fenpyroximate	pyrazole insecticide

20	96489-71-3	Pyridaben	Pyridaben	miticide/insecticide
21	85-01-8	Phenanthrene	Phenanthrene	PAH
22	78-48-8	Tributyl phosphorotrithioate	Tribufos	OP pesticide
23	122-39-4	Diphenylamine	Diphenylamine	Plant growth regulator; N containing
24	120-12-7	Anthracene	Anthracene	PAH
25	153233-91-1	Etoxazole	Etoxazole	miticide
26	129-00-0	Pyrene	Pyrene	PAH
27	101-20-2	Triclocarban	3,4,4'-Trichlorocarbanilide	antimicrobial in personal care products
28	56-72-4	Coumaphos	Coumaphos	OP pesticide
29	2104-64-5	EPN	O-Ethyl O-(p-nitrophenyl) phenylphosphonothioate	OP pesticide
30	62-38-4	Phenylmercuric acetate	Phenylmercuric acetate	organic mercury salt
31	191906	Captafol	Captafol	fungicide
32	72-55-9	DDE	p,p'-DDE	OC metabolite
33	42874-03-3	Oxyfluorfen	Oxyfluorfen	herbicide
34	2058-94-8	Henicosafluoroundecanoic acid	Perfluoroundecanoic acid	perfluroalkyls; fluorosurfactant
35	309-00-2	Aldrin	Aldrin	OC pesticide
36	143-50-0	Kepone	Kepone (Chlordecone)	OC pesticide
37	1836-75-5	Nitrofen	2,4-dichloro-1-(4-nitrophenoxy)benzene	alkyl halide
38	NOCAS_47366	Pharma4736	5-fluoro-1-(3-fluorobenzyl)-N-(1H-indol-5-yl)-1H-indole-2-carboxamide	failed pharmaceutical
39	115-29-7	Endosulfan	Endosulfan	OC pesticide
40	173584-44-6	Indoxacarb	Indoxacarb	oxadiazine insecticide
41	76-44-8	Heptachlor	Heptachlor	OC pesticide
42	335-76-2	Perfluorodecanoic acid	Perfluorodecanoic acid	perfluroalkyls; fluorosurfactant
43	2795-39-3	Potassium perfluorooctanesulfonate	Heptadecafluorooctanesulfonic acid potassium salt	perfluroalkyls; fluorosurfactant

44	115-32-2	Dicofol	Dicofol	OC pesticide
45	7487-94-7	Mercuric chloride	Mercuric chloride	inorganic mercury salt
46	754-91-6	Perfluorooctanesulfonamide	Perfluorooctanesulfonamide	perfluroalkyls; fluorosurfactant
47	57-74-9	Chlordane	Chlordane	OC pesticide
48	55285-14-8	Carbosulfan	Carbosulfan	carbamate
49	95737-68-1	Pyriproxyfen	Pyriproxyfen	pyridine pesticide (fleas)
50	56-55-3	Benz[a]anthracene	Benz[a]anthracene	PAH

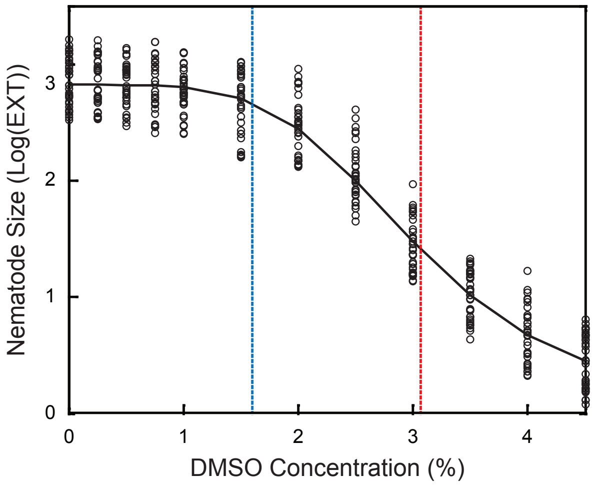

Figure S1. Effect of DMSO (vehicle control) on *C. elegans* growth. The red dashed line indicates the AC_{50} (half-maximal concentration; 3.1 % DMSO); the blue dashed line indicates the benchmark concentration lower limit (1.6 % DMSO), below which no effect was observed compared to the untreated control. The benchmark concentration was calculated using mean nematode sizes for exposure wells, with size at time = 0 subtracted from the size at t=48 h. The definition of Crump (2002) was used to calculate the BMC with bootstrapping on residuals for the 90% one-sided confidence interval as previously described (Boyd et al. 2010a; Crump 2002).
